# A novel data-driven workflow combining literature and electronic health records to estimate comorbidities burden for a specific disease: a case study on autoimmune comorbidities in patients with celiac disease

**DOI:** 10.1186/s12911-017-0537-y

**Published:** 2017-09-29

**Authors:** Jean-Baptiste Escudié, Bastien Rance, Georgia Malamut, Sherine Khater, Anita Burgun, Christophe Cellier, Anne-Sophie Jannot

**Affiliations:** 1 Georges Pompidou European Hospital (HEGP), AP-HP, Paris, France; 2 INSERM UMRS 1138, Paris Descartes University, Paris, France; 3 Pôle Informatique Médicale et Santé Publique, Hôpital Européen Georges Pompidou, 20 rue Leblanc, 75015 Paris, France

**Keywords:** Autoimmune diseases, Celiac disease, Electronic health records, Icd 10, Phenotype, Prevalence study, Diabetes mellitus, type 1, Dermatitis herpetiformis, Thyroiditis, autoimmune, Arthritis, rheumatoid, Lupus erythematosus, systemic, Multiple sclerosis, Sjogren’s syndrome, Addison disease, Arthritis, juvenile, Hepatitis, autoimmune, Graves’ disease, Myasthenia gravis, Polyendocrinopathies, autoimmune, Antiphospholipid syndrome

## Abstract

**Background:**

Data collected in EHRs have been widely used to identifying specific conditions; however there is still a need for methods to define comorbidities and sources to identify comorbidities burden. We propose an approach to assess comorbidities burden for a specific disease using the literature and EHR data sources in the case of autoimmune diseases in celiac disease (CD).

**Methods:**

We generated a restricted set of comorbidities using the literature (via the MeSH® co-occurrence file). We extracted the 15 most co-occurring autoimmune diseases of the CD. We used mappings of the comorbidities to EHR terminologies: ICD-10 (billing codes), ATC (drugs) and UMLS (clinical reports). Finally, we extracted the concepts from the different data sources. We evaluated our approach using the correlation between prevalence estimates in our cohort and co-occurrence ranking in the literature.

**Results:**

We retrieved the comorbidities for 741 patients with CD. 18.1% of patients had at least one of the 15 studied autoimmune disorders. Overall, 79.3% of the mapped concepts were detected only in text, 5.3% only in ICD codes and/or drugs prescriptions, and 15.4% could be found in both sources. Prevalence in our cohort were correlated with literature (Spearman’s coefficient 0.789, *p* = 0.0005). The three most prevalent comorbidities were thyroiditis 12.6% (95% CI 10.1–14.9), type 1 diabetes 2.3% (95% CI 1.2–3.4) and dermatitis herpetiformis 2.0% (95% CI 1.0–3.0).

**Conclusion:**

We introduced a process that leveraged the MeSH terminology to identify relevant autoimmune comorbidities of the CD and several data sources from EHRs to phenotype a large population of CD patients. We achieved prevalence estimates comparable to the literature.

**Electronic supplementary material:**

The online version of this article (10.1186/s12911-017-0537-y) contains supplementary material, which is available to authorized users.

## Background

Precise phenotyping of patient data remains one of the key points of personalized medicine. From a clinical perspective, detailed knowledge of the comorbidities enables targeted treatment strategies. And from a research perspective, specific and accurate phenotypes allow the recruitment of patients in observational or interventional studies. Clinical Data Warehouses (CDW) gather information on hundred thousands of patients. These CDWs can be used to phenotype comorbidities as in our institution [1].

To phenotype patients using Electronic Health Records (EHRs), many different EHR sources could be mined: for instance International Classification of Diseases (ICD) codes, clinical reports, drug prescriptions, procedures, and laboratory test results if relevant. ICD codes have been widely used to phenotype patients [2]. Several studies [3, 4] showed that billing codes were specific enough to identify patients suffering from a given condition such as inflammatory bowel disease. However, ICD codes have a low sensitivity, particularly if the main claim was another condition, because then the studied condition is less likely to be coded. Therefore, ICD codes might perform poorly to phenotype comorbidities. The clinical narrative within the EHRs forming the patient’s medical history contains lots of detailed information such as data collected outside the hospital, e.g., results of lab tests performed before the admission, or information regarding decision support, e.g., rejected clinical hypotheses. Disorder terms are indeed present in various types of clinical narratives, such as radiology reports [5, 6], medical observations [7, 8], nurse narratives [9]– and generally every document produced for healthcare activity. Several authors reported that clinical notes offer good sensitivity; moreover combining billing codes, clinical notes, and medications provides superior phenotyping performance [2, 10–12].Strategies such as phenotyping algorithms combining different types and sources of data (e.g. as proposed by PheKB [13]) are promising, but they require expert time to develop and the number of available algorithms are limited and focused on specific diagnoses.

Celiac disease (CD) is an autoimmune disorder triggered by gluten in genetically susceptible individuals. The disease is characterized by autoantibodies directed against gluten such as anti-gliadin or other targets (anti-transglutaminase, anti-endomysial). Many symptoms can be associated with CD, the most prominent are caused by malabsorption. CD is also associated with numerous autoimmune comorbidities. These comorbidities add to the high burden of symptoms and complications for these patients, and might be target for specific treatment strategies and screening programs. This is why it is necessary to identify these subpopulations for clinical research and public health policies. Previous epidemiological studies have shown that dysthyroidism and type 1 diabetes mellitus were the most prevalent diseases in patients with CD (6.0 to 30.2% and 2.2 to 6.5% respectively) [14–20]. Several methods were used, namely autoantibody detection [17–20], questionnaires [14, 15, 21, 22], national register [23] and retrospective reviews of medical records [16, 24], providing heterogeneous results, and only estimates for one or few autoimmune comorbidities. The estimated prevalence of thyroid disorders varies largely in these studies (from 6 to 30%). Other authors have studied the prevalence of CD among patients with autoimmune diseases [25]. For example, there is a high prevalence of CD in young diabetic patients [26].

To the best of our knowledge, there is no clear review on the most prevalent set of autoimmune comorbidities associated to CD, while there is a need to phenotype autoimmune comorbidities burden in CD patients to enable further stratification of patients’ profiles. While methods to phenotype patients for a specific disease exists, they do not allow to estimate comorbidities burden in a specific domain. This step requires usually domain experts to define a set of specific comorbidities, but this elicitation step might introduce bias. Biomedical literature and its metadata can also be mined to extract knowledge for various purposes [27]: precision medicine and drug repositioning, pathway extraction, gene function prediction, data integration, pharmacogenomics, toxicology. As the biomedical literature also explores comorbidities associated to diseases, its metadata could provide information on relevant comorbidities for a given disease.

The present study aimed to show how a workflow based on both literature and EHRs information can help identifying relevant autoimmune comorbidities in CD and to phenotype for these autoimmune comorbidities the population of adult CD patients followed up in Georges Pompidou European Hospital (HEGP). We assessed its performance in this context by assessing quantitatively whether literature-based knowledge was correlated to knowledge extracted from EHRs regarding autoimmune CD comorbidities. Our secondary objective aimed at assessing to what extent three major EHR components (ICD codes, drug prescriptions and narrative reports) contributed to identifying comorbidities in the specific domain of autoimmune comorbidities in our population.

## Methods

### Overview

We first selected the list of autoimmune diseases from MeSH® terminology. We then restricted this list to the most frequent autoimmune diseases associated with CD in the literature, based on the number of co-occurrences of MeSH® terms in MEDLINE®. We mapped selected autoimmune disorders to different terminologies to identify concepts and terms for data collection. Then we included CD patients from a local registry and completed by querying the hospital clinical data warehouse (CDW). Finally we identified status for selected autoimmune diseases for these patients by reviewing their EHR for mapped concepts.

### Selection of a restricted set of autoimmune diseases using MeSH® co-occurrence file

To define autoimmune comorbidities burden in CD, we first extract a specific set of autoimmune disease. An initial list of autoimmune diseases was defined as: all the children of concept ‘Autoimmune Disease’ (*D001327*) in the MeSH® hierarchy, i.e., all the descriptors with a MeSH® *Tree Number* starting with *C20.111*. We identified amongst this list the autoimmune diseases most frequently associated with CD in MEDLINE® using the MeSH® co-occurrence file (MRCOC) provided by the U.S. National Library of Medicine as part of the UMLS Metathesaurus (2014AB release). The MRCOC file contains the number of times each pair of MeSH® descriptors occurred in MEDLINE® citations. We extracted all pairs of MeSH terms that contained both the MeSH descriptor for *Celiac Disease (D002446)* and any MeSH descriptor for autoimmune diseases. We arbitrarily restricted the list for this study to the 15 autoimmune diseases the most co-occurring with CD. This process allows to focus on a domain of comorbidities using the MeSH hierarchy and to a subset of comorbidities using the number of co-occurrences in MEDLINE®.

### MeSH® mapping to drugs and diagnoses terminologies

We used the Unified Medical Language System (UMLS) to map autoimmune disorders MeSH® concepts to other terminologies used in the CDW. To leverage diagnosis codes we used the International Classification of Diseases version 10 (ICD-10). We used the Anatomical Therapeutic Chemical Classification System (ATC) whenever a drug was specific to an autoimmune disease. These terminologies (MeSH, ICD-10 and ATC) also provided terms to constitute a catalog of words for helping textual data review.

### Study population

The HEGP hospital has a specialized consultation for CD patients with about 50 new patients recruited per year in the last decade. The gastroenterology department has maintained a list of patients with CD for research purposes. The study has been approved and data access granted by the following commissions: the *“Commission Nationale de l’Informatique et des Libertés”* (declaration #174350) and the *HEGP institutional review board* (registration #00001072).

To complete the list of patients maintained by the gastroenterology department, we also extracted patients fulfilling the three following criteria: (i) at least one encounter in the 2000-2014 period of time (ii) presence of at least one ICD 10 code for CD (K90) in billing claims; (ii) one or more hospitalization stay or consultation in the gastroenterology department and (iii) at least one text document (discharge or letter) containing the term ‘celiac disease’ or one of its synonyms. We extracted these data from HEGP i2b2 CDW [25]. This CDW contains routine care data divided into several categories among them demographics (age, sex, and vital status), vital signs (e.g., temperature, blood pressure, weight…), diagnoses (ICD-10), procedures (French classification), clinical data (structured questionnaires from EHR), free text reports, pathology codes (French ADICAP classification), biological test results (LOINC), and Computerized Provider Order Entry (ATC) drug prescriptions [28].

**Fig. 1:**
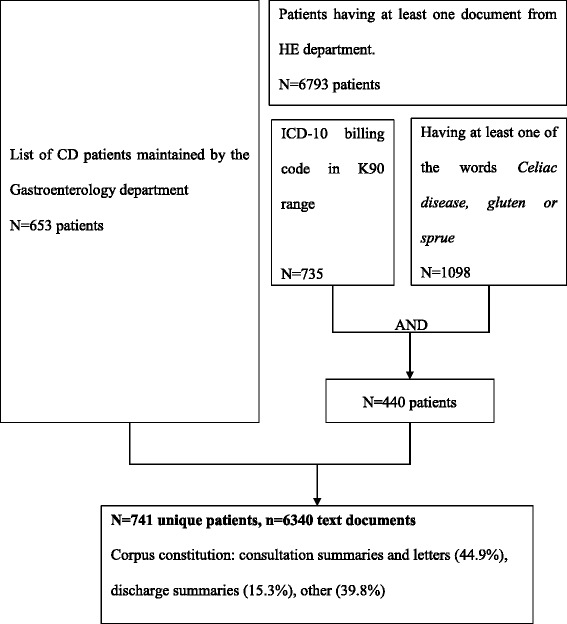
Patients inclusion flowchart

The study population counted 741 patients and a corpus of 6340 clinical reports (patients’ inclusion flowchart available in Fig. 1).

### Autoimmune diseases phenotyping

We queried the CDW to identify for each patient the presence/absence of the selected autoimmune diseases. We used the selected ICD-10 diagnosis codes to query billing data and selected ATC codes to query Computerized Prescription Order Entry data.

We extracted every narrative report including discharge summaries, outpatient reports, multi-disciplinary expert meeting summaries and letters from all departments of the hospital and reviewed them manually to extract selected autoimmune disease status for each patient. To facilitate the manual review, we developed a browser-accessible software, FASTVISU [29], interfaced with the CDW to display and explore all the documents for a given patient. FASTVISU highlights terms with an entity recognition module based on a list of regular expressions (e.g., the pattern \bdiab\w + \b to match for diabetes) defined by the user and approximate matching techniques. For the 15 selected autoimmune comorbidities, a set of regular expressions was established, and used to query the CDW and obtain the CD corpus; then two trained physicians reviewed all the clinical narratives in the CD corpus using FASTVISU to validate the presence/absence of each of the selected autoimmune diseases for each patient.

Because autoimmune diseases are chronic diseases, a patient was considered having the autoimmune disorder if the condition was ascertained at least once in her/his EHR. The analysis was performed considering all sources together then each source separately.

**Fig. 2:**
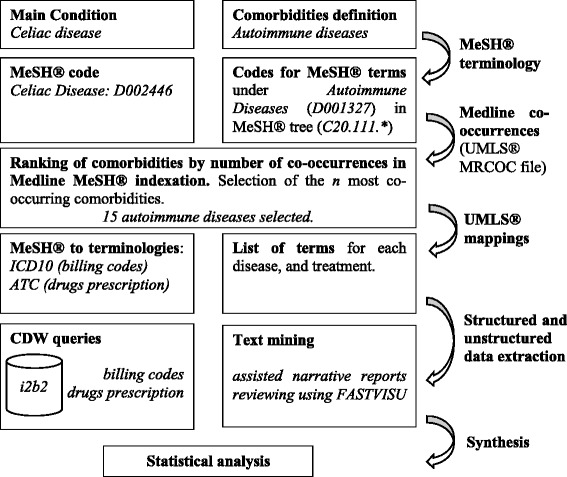
Workflow from comorbidities selection to comorbidities burden phenotyping

Figure 2 illustrates our workflow.

### Statistical analysis

Patients’ characteristics were summarized using median and interquartile ranges for quantitative variables and proportions for qualitative variables. We measured Sperrin’s *I* coefficient [30] to evaluate temporal irregularity of the data recorded in the EHR, denoting whether some encounters would provide more data and, consequently, whether other time periods would be less covered. Sperrin’s *I* coefficient was calculated for each patient with at least 2 encounters using formula (1):

1 $$ I\kern0.5em =\kern0.5em \frac{2}{n}\kern0.5em +\kern0.5em \frac{n-2}{n}\kern0.5em \left[1-\sqrt{\left(n-1\right)\kern0.5em Var\left({g}_i;\kern0.5em i\kern0.5em =\kern0.5em 1,\dots, \kern0.5em n-1\right)}\right] $$

Reviewers’ mutual-agreement in the text review was evaluated with Cohen’s alpha coefficient.

For each autoimmune disease, a case was defined by at least one ICD code, one drug prescription, or one mention in text. For each prevalence estimate, we computed the 95% confidence interval or Wilson’s interval if the proportion was close to 0%.

To compare the contributions of text, ICD codes and drugs to estimate autoimmune comorbidities, we computed the proportions of cases detected by text alone, structured information (ICD code or drug prescription on CPOE) alone, or both.

Finally, to assess the performance of literature-based comorbidities selection, we estimated the correlation between MeSH® and diseases prevalence, using Spearman’s correlation coefficient between number of publications indexed with MeSH® terms for both CD and an autoimmune disease, and the prevalence the corresponded disease obtained from our EHR extraction.

Statistical analysis was conducted with R (version 3.1.2).

## Results

We selected the first fifteen autoimmune disease whose MeSH® terms co-occurred the most with CD MeSH® terms in MEDLINE (Table 1). The most frequent were type I diabetes (523 citations), dermatitis herpetiformis (478 citations), and thyroiditis (96 citations). The number of co-occurrences ranged from 12 to 523.

**Table 1:** Autoimmune disease selection by descending order of co-occurrence frequencies and ICD-10 codes used for diagnosis extraction

MeSH® terms	N co-occurrences	DUI^*^	ICD-10
Diabetes Mellitus, Type 1	523	D003922	E10.^a^
Dermatitis Herpetiformis	478	D003874	L130
Thyroiditis, Autoimmune	96	D013967	E063
Arthritis, Rheumatoid	87	D001172	M069.^a^
Lupus Erythematosus, Systemic	73	D008180	M32.^a^
Multiple Sclerosis	44	D009103	G35
Sjogren’s Syndrome	43	D012859	M350
Addison Disease	42	D000224	E271|E272
Arthritis, Juvenile	37	D001171	M089.^a^
Hepatitis, Autoimmune	35	D019693	K754
Graves’ Disease	30	D006111	E050|E05.^a^
Glomerulonephritis, IGA	27	D005922	N0330|N0170
Myasthenia Gravis	22	D009157	G700
Polyendocrinopathies, Autoimmune	15	D016884	E31.^a^
Antiphospholipid Syndrome	12	D016736	D686.^a^

* DUI Descriptor Unique Identifiers

^a^Means all descending nodes, | means OR

The mapping of the selected autoimmune diseases to ICD-10 provided 39 diagnosis codes (Table 1). For the mapping of the selected disease to ATC, levothyroxine was used as marker for dysthyroidism, and insulin as a marker for type I diabetes. Insulin and levothyroxine corresponded to a total of 263 ATC codes (Additional file 1). We built a catalog of 55 terms from ICD-10, ATC and MeSH to help review of narratives.

The 741 included patients had overall 6340 clinical reports. Patients’ characteristics are presented in Table 2. Ages spanned adulthood, with a mean of 42.5 years and a standard deviation of 16.9 years. Most were female, with a sex ratio of 2.8.Eighteen patients (2.4%) had only one encounter. One third of the patients had been followed up for 5 to 20 years. Patients had a median of 5 [3; 10] clinical documents, with a maximum of 146. Half of the CD patients had no other ICD-10 code than CD, 19.7% had 1 to 5 distinct ICD-10 codes, and 12.7% had between 6 and 69 codes. Most patients (93.5%) had at least one hospitalization stay during the 2000-2014 period, as all patients are offered a day hospital admission for initial management of their CD.

**Table 2:** Population characteristics

Age in years, mean (SD)	42.5 (16.9)
Sex, n (%)	
Female	545 (73.6)
Male	196 (26.4)
Follow-up time in years per patient, n (%)	
0 (1 encounter)	18 (2.4)
0–1	248 (33.5)
1–2	91 (12.3)
2–5	134 (18.1)
5–20	250 (33.7)
In- or Outpatient, n (%)	
Outpatients	48 (6.5)
Inpatients	693 (93.5)
Number of encounters, median (IQR), max	3 (2, 4), 47
Documents per patient, median (IQR), max	5 (3, 10), 146
Sperrin’s I irregularity indicator, mean (SD), *n* = 638	0.759 (0.10)
Number of distinct ICD-10 codes per patient, n (%) ^a^	
0	372 (50.2)
1	129 (17.4)
2–5	146 (19.7)
6–69	94 (12.7)

*IQR* interquartile range

^a^Except code K900 for Celiac disease

Sperrin’s *I* coefficient was 0.759 mean (SD) 0.10, indicating that patients were followed up regularly.

Readers voted on 466 items. More specifically, 465 patients out of the 741 included patients had no highlighted terms and 466 autoimmune disease items had at least one highlighted term occurrence on the 276 remaining patients. For 140 items, voters both approved that the patient suffered from this disease; for 304 items, readers both disapproved that the patient suffered from this disease and for 22 items, readers mutually disagreed. Therefore, inter-reviewer agreement for autoimmune disorder identification in narrative reports was excellent, with a Cohen’s kappa value of 0.89.

The prevalence estimates of the 15 selected autoimmune diseases with their 95% confidence intervals are presented in Tables 3 and 4 together with literature estimates.

**Table 3:** Prevalence estimates

Disease	N cases	Prevalence per 1000 patient [CI95]
Autoimmune thyroiditis	93	125.5 (101; 149)
Type 1 diabetes mellitus	17	22.9 (12.1; 33.5)
Dermatitis herpetiformis	15	20.2 (10; 30.2)
Rheumatoid arthritis	7	9.4 (2.5; 16.3)
Autoimmune hepatitis	7	9.4 (2.5; 16.3)
Graves’ disease	6	8.1 (1.6; 14.5)
Sjogren’s syndrome	6	8.1 (1.6; 14.5)
Addison disease	3	4 (1.4; 11.8)
Systemic lupus erythematosus	2	2.7 (0.7; 9.8)^a^
Juvenile arthritis	1	1.3 (0.1; 7.6)^a^
Multiple sclerosis	1	1.3 (0.1; 7.6)^a^
Autoimmune Polyendocrinopathies	1	1.3 (0.1; 7.6)^a^
Antiphospholipid syndrome	0	0 (0; 5.2)^a^
Myasthenia gravis	0	0 (0; 5.2)^a^

CI95: 95% confidence interval

^a^Wilson’s intervals

**Table 4:** Comparison with prevalence in the literature

	This study	Cosnes et al. [33]	Iqbal et al. [2]	Volta et al. [5]	Collin et al. [22]	Størdal et al. [6]	Diamanti et al. [1]	Van der Pals et al. [13]	Sategna-Guidetti et al. [27]	Counsell et al. [34]
Study characteristics										
Population	741 adults	378 children, 546 adults	356 adults (> 12 yo)	770 adults	335 adults	3006 children	558 children	335 children	241 adults (untreated)	107 adults
Information source	EHR	question-naire	question-naire	question-naire	hospital records	national register	serology	serology	serology	serology
Origin	France	France	Canada	Italy	Finland	Norway	Italy	Sweden	Italy	Scotland
Year	2015	2008	2013	2012	1994	2011	2011	2014	2001	1994
Prevalence, % (95% CI)										
Autoimmune thyroiditis	12.6 (10.1, 14.9)	6.0	10.6	26.3	7.5	1.4	12.0	7.7	30.2	15.0
Type 1 diabetes mellitus	2.3 (1.2, 3.4)	6.5	2.2	3	5.4	4.7				
Dermatitis herpetiformis	2.0 (1.0, 3.0)	3.1	13.5	4						
Rheumatoid disease	0.9 (0.3, 1.6)	0.7	4.5		1.8					
Autoimmune hepatitis	0.9 (0.3, 1.6)	1.2	0.0							
Sjögren’s syndrome	0.8 (0.2, 1.5)	0.2	0.8		3.3					
Addison’s disease	0.4 (0.1, 1.2)	0.2			0.6					
Systemic lupus	0.3 (0.1, 1.0) ^a^	0.2	1.1							
Multiple sclerosis	0.1 (0.0, 0.8) ^a^	0.1								
Antiphospholipid syndrome	0 (0.0, 0.5) ^a^	0.2								
Myasthenia gravis	0 (0.0, 0.5) ^a^	0.2								

CI95: 95% confidence interval, ^*a*^ Wilson’s interval

**Fig. 3:**
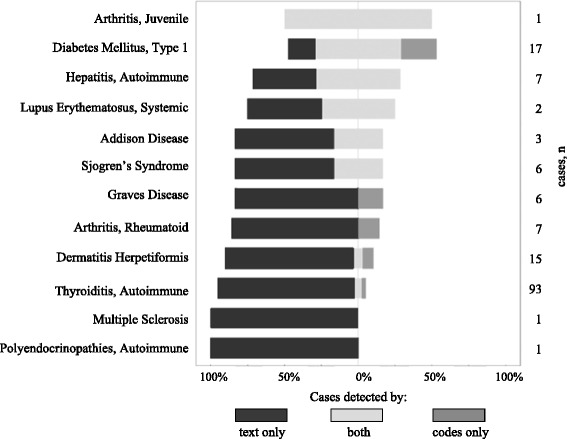
Phenotypes identified by text reviewing only (black), ICD codes or drugs only (grey), both (light grey), in percent

Figure 3 represents the respective contributions of text and structured information to identify cases. Overall, 79.3% of the cases were detected only in text, 5.3% only in ICD codes and/or drugs prescriptions, and 15.4% could be found in both types of sources, with variations across the diseases. 86% of dermatitis herpetiformis diagnoses were mentioned only in narrative reports, whereas only 7% were found as structured information exclusively; in 7% of the cases the dermatitis herpetiformis information was present in both text and ICD codes. Information regarding autoimmune thyroiditis was mostly present only in text (92.5% of detected cases), with only 2.2% of cases detected in codes alone, and 5.3% in both text and structured data (ICD code and/or drugs prescription). Type 1 diabetes was detected by text alone in 17.6% of the cases, codes alone in 23.5%, and found in both in 58.8% of the cases.

The three most prevalent diseases were thyroiditis (12.6%), type 1 diabetes (2.3%) and dermatitis herpetiformis (2.0%). 18.1% of CD patients had at least one of the 15 autoimmune diseases, in accordance with literature estimates. Prevalence estimates strongly correlate with co-occurrence ranking for the 15 autoimmune diseases, as measured by a Spearman’s correlation coefficient value of 0.789 (*P* value = 0.0005). The three most prevalent autoimmune diseases in our adult CD population appeared as the top three in the co-occurrence ranking.

## Discussion

We successfully identify major CD autoimmune comorbidities using a novel data-driven workflow leveraging MeSH® terminology and Medline MeSH® co-occurrences. We mapped these comorbidities to terminologies used in EHRs to phenotype a new set of 741 patients achieving prevalence estimates comparable to the literature. One finding is the importance of clinical text reports as the most informative data source to phenotype patients was clinical narratives.

### External validation of prevalence estimates

The mining of EHR data allowed us to include 741 patients, one of the largest population of adult CD patients used to report autoimmune diseases prevalence in CD patients to the best of our knowledge. In this hospital based study, the prevalence of autoimmune comorbidity was 18.1% (95% CI 15.4 –21.0). The three most prevalent autoimmune comorbidities were thyroiditis with a prevalence of 12.6% (95% CI 10.1–14.9), type 1 diabetes mellitus with 2.28% (1.2 –3.4) and dermatitis herpetiformis with 2.0% (95% CI 1.0–3.0).

We compared our prevalence estimates with literature as an external validation. Our disease prevalence estimates are in the highest range compared to other published studies [14–20, 23]. The first explanation is that we assessed prevalence from a hospital-based cohort from a CD specialized center, while most non-complicated CD disease, therefore with no additional autoimmune burden, are likely to be followed-up in ambulatory care only. Moreover, our study benefited from the coverage of the CDW, which includes a long follow-up and, therefore, increases the probability of mentioning an associated autoimmune disease. The quality of this longitudinal source of information was measured by Sperrin’s coefficient, which demonstrates a broad coverage of text documents during the follow-up period. In contrast, prevalence studies based on questionnaires [14, 15, 21] may underestimate prevalence, e.g., due to memory bias.

It would have been of interest to extract diagnosis date, but as we expected many missing data and approximations due to early childhood diagnoses, we did not extract this information.

### Text reports were more sensitive than ICD codes and medications for detecting autoimmune comorbidities

In this study, most diagnoses were ascertained through text reports. This finding is consistent with the review by Shivade et al. of 97 studies using EHR for phenotype identification [2]. A typical example is thyroiditis, where about 98% of the cases were found in the text reports (92.5% only in text, and 5.3% in both text and structured data).

Few diagnoses cases were ascertained through ICD-10 codes. In France, as in many countries, ICD-10 coding is primarily used for billing purposes and limited to inpatients. Consequently, the coding does not aim to cover all the patient’s conditions [31]. Moreover narrative reports include extensive information such as a dedicated *medical history* section [10]. Additionally, autoimmune disease cases were identified in our study using documents produced during outpatient visits during which no ICD-10 codes were collected in line with French regulation (no ICD-10 codes are produced during outpatient visits in France). Similarly, Wei et al. analyzed the respective contributions of clinical notes, ICD codes and medications for detecting ten diseases in EHRs and showed that clinical narratives offered the best sensitivity (0.77) [12].

Our results showed the benefits of combining text mining and structured data extraction. Other examples are found in the literature in colon cancer [11], atrial fibrillation, Alzheimer’s disease, breast cancer, gout, human immunodeficiency virus infection, multiple sclerosis, Parkinson’s disease, rheumatoid arthritis, and types 1 and 2 diabetes mellitus [12].

### Literature-based selection of autoimmune diseases

To the best of your knowledge there was no clear synthesis of major CD autoimmune comorbidities. The novel approach mining literature presented in this study enabled to identify relevant comorbidities as attested by the fact that the attention from the literature was coherent with the prevalence found both in the literature and in our cohort: autoimmune dysthyroidism or type 1 diabetes mellitus appeared in the top co-occurring MeSH® terms and these comorbidities were described in the literature as being the most prevalent autoimmune comorbidities in CD patients [16, 20]. Furthermore, the three most prevalent autoimmune diseases in our adult CD population appeared as the top three in the co-occurrence ranking. Our method is flexible as domain restriction using MeSH® hierarchy and limiting the number of results with the number of co-occurrence are both optional, although we haven’t evaluated this method without these two types of restrictions. Moreover, this novel approach provided us with the most recent list of autoimmune diseases associated with CD in the literature. This is of interest because research subjects evolve over time and in this light Medline acts as a biomedical research collective memory and an up-to-date view of clinical expertise. For example, based on MEDLINE co-occurrences before year 2000, *Autoimmune Hepatitis* would not be in the top 15 selection, but *Pemphigus* and *Pemphigoid, Bullous* would be (see Additional file 2). Combined with EHR mining give us prevalence estimate of comorbidities that were not suspected as being associated to CD at the time patients’ diagnoses were recorded. Another advantage of this data-driven selection is to provide an automatable alternative to the usual elicitation step which classically determines relevant comorbidities by domain experts. This method allows to design more pragmatic studies, not relying on one or two experts’ opinion.

### Automatable and RECORD statement compliant workflow

The workflow of this study, i.e. comorbidities selection from the MeSH® co-occurrences file, mapping from MeSH® to other terminologies, and case identification through text and coded data mining, can be automated to estimate comorbidities burden in other EHR-based studies.

Our workflow is in line with RECORD statement [32], in particular reporting a complete list of codes and algorithms for each comorbidities.

Moreover, we demonstrated that manual review could be performed easily using text visualization tools integrated with the CDW, even for non-English language based EHR [29].

Phenotyping quality is sometimes considered as a limit of EHR reuse. In the proposed workflow, we reinforce phenotyping quality by manual phenotype extraction by two readers in reasonable time thanks to assisted extraction using a visualization tool, FASTVISU. While FASTVISU is based on regular expressions which lack of precision, our workflow could be improved using a natural language based tool.

## Conclusion

We provide an automatable workflow fulfilling requirements from RECORD statement on observational routinely-collected health data aiming at identifying comorbidities burden for a specific disease using EHR. We applied this workflow to finely phenotype autoimmune comorbidities in a large CD population. We think that this flexible workflow will ease the extraction of relevant information from EHR.

## Additional files

Additional file 1: List of ATC codes used. Lists of Anatomical Therapeutic Chemical Classification System (ATC) codes used for autoimmune thyroiditis (levothy*) and for diabetes mellitus, Type 1 (insulin). (DOCX 13 kb)

Additional file 2: Evolution of the numbers of co-occurrences in time. The 15 first ranked autoimmune diseases (in red) which would have been included based on the literature available at various time points. Numbers of co-occurrences until the specified year, ranks in prevalence estimates from this study, ranks in number of MeSH terms co-occurrence with term ‘Celiac Disease’ in MEDLINE at specified years. First version of the clinical vignette related on a new analgesic to control pain in mild trauma injuries with the four experimental factors tested. Description of first clinical vignette and list of response options. (DOCX 17 kb)
