# Supplementary material for: A novel data-driven workflow combining literature and electronic health records to estimate comorbidities burden for a specific disease: a case study on autoimmune comorbidities in patients with celiac disease
Source: BMC Med Inform Decis Mak. 2017 Sep 29;17:140. doi: 10.1186/s12911-017-0537-y (PMC5622531; doi:10.1186/s12911-017-0537-y)
Supplement: Supplementary file 1 — List of ATC codes used. Lists of Anatomical Therapeutic Chemical Classification System (ATC) codes used for autoimmune thyroiditis (levothy*) and for diabetes mellitus, Type 1 (insulin). (DOCX 13 kb) [file 12911_2017_537_MOESM1_ESM.docx]

**ANNEX 2** – Levothyroxine was used as marker for dysthyroidism insulin for type 1 diabetes. This table lists all Anatomical Therapeutic Chemical Classification System (ATC) codes for terms containing “*levothy”* and ATC codes A10A (insulin) and its children.

| **Autoimmune disease** | **ATC codes** |  |
| --- | --- | --- |
| Thyroiditis, Autoimmune (*levothy**) | H03AA01, H03AA03 |  |
| Diabetes Mellitus, Type 1 (*insulin*) | A10AB01, A10AB02, A10AB03, A10AB04, A10AB05, A10AB06, A10AB30, A10AC01, A10AC02, A10AC03, A10AC04, A10AC30, A10AD01, A10AD02, A10AD03, A10AD04, A10AD05, A10AD06, A10AD30, A10AE01, A10AE02, A10AE03, A10AE04, A10AE05, A10AE06, A10AE30, A10AF01 |  |
