# Supplementary material for: A novel data-driven workflow combining literature and electronic health records to estimate comorbidities burden for a specific disease: a case study on autoimmune comorbidities in patients with celiac disease
Source: BMC Med Inform Decis Mak. 2017 Sep 29;17:140. doi: 10.1186/s12911-017-0537-y (PMC5622531; doi:10.1186/s12911-017-0537-y)
Supplement: Supplementary file 2 — Evolution of the numbers of co-occurrences in time. The 15 first ranked autoimmune diseases (in red) which would have been included based on the literature available at various time points. Numbers of co-occurrences until the specified year, ranks in prevalence estimates from this study, ranks in number of MeSH terms co-occurrence with term ‘Celiac Disease’ in MEDLINE at specified years. First version of the clinical vignette related on a new analgesic to control pain in mild trauma injuries with the four experimental factors tested. Description of first clinical vignette and list of response options. (DOCX 17 kb) [file 12911_2017_537_MOESM2_ESM.docx]

**ANNEX 1** – The 15 first ranked autoimmune diseases (in red) which would have been included based on the literature available at various time points. Numbers of co-occurrences until the specified year, ranks in prevalence estimates from this study, ranks in number of MeSH terms co-occurrence with term ‘*Celiac Disease*’ in MEDLINE at specified years.

| **MeSH TERM** | **N co-occurrences 2014** | **Prevalence Rank** |  | **2014** | **2009** | **2004** | **2000** |
| --- | --- | --- | --- | --- | --- | --- | --- |
| Diabetes Mellitus, Type 1 | 523 | 2 |  | 1 | 2 | 2 | 2 |
| Dermatitis Herpetiformis | 478 | 3 |  | 2 | 1 | 1 | 1 |
| Autoimmune Diseases | NA | NA |  | NA | NA | NA | NA |
| Thyroiditis, Autoimmune | 96 | 1 |  | 3 | 3 | 5 | 6 |
| Arthritis, Rheumatoid | 87 | 4.5 |  | 4 | 4 | 3 | 3 |
| Lupus Erythematosus, Systemic | 73 | 9 |  | 5 | 5 | 5 | 4 |
| Multiple Sclerosis | 44 | 11 |  | 6 | 8 | 8 | 10 |
| Sjogren's Syndrome | 43 | 6.5 |  | 7 | 6 | 6 | 5 |
| Addison Disease | 42 | 8 |  | 8 | 7 | 7 | 8 |
| Arthritis, Juvenile | 37 | 11 |  | 9 | 11 | 10 | 7 |
| Hepatitis, Autoimmune | 35 | 4.5 |  | 10 | 11 | 14 | 19 |
| Graves’ Disease | 30 | 6.5 |  | 11 | 11 | 11 | 12 |
| Glomerulonephritis, IGA | 27 | 14 |  | 12 | 9 | 9 | 10 |
| Myasthenia Gravis | 22 | 14 |  | 13 | 13 | 11 | 10 |
| Polyendocrinopathies, Autoimmune | 15 | 11 |  | 14 | 15 | 15 | 15 |
| Antiphospholipid Syndrome | 12 | 14 |  | 15 | 15 | 17 | 19 |
| Pemphigus |  |  |  | 16 | 14 | 13 | 13 |
| Purpura, Thrombocytopenic, Idiopathic |  |  |  | 17 | 17 | 19 | 19 |
| Anemia, Hemolytic, Autoimmune |  |  |  | 18 | 17 | 17 | 16 |
| Neuromyelitis Optica |  |  |  | 19 | 25 | 32 | 30 |
| Pemphigoid, Bullous |  |  |  | 19 | 19 | 16 | 14 |
| Autoimmune Diseases of the Nervous System |  |  |  | 21 | 20 | 26 | 39 |
| Glomerulonephritis, Membranous |  |  |  | 22 | 21 | 21 | 23 |
| Granulomatosis with Polyangiitis |  |  |  | 23 | 25 | 21 | 23 |
| Anti-Glomerular Basement Membrane Disease |  |  |  | 27 | 33 | 26 | 23 |
| Churg-Strauss Syndrome |  |  |  | 27 | 33 | 26 | 38 |
| Dysautonomia, Familial |  |  |  | 27 | 25 | 21 | 19 |
| Giant Cell Arteritis |  |  |  | 27 | 25 | 21 | 19 |
| Guillain-Barre Syndrome |  |  |  | 27 | 33 | 37 | 35 |
| Stiff-Person Syndrome |  |  |  | 27 | 25 | 31 | 29 |
| Vasculitis, Central Nervous System |  |  |  | 27 | 25 | 26 | 26 |
| Demyelinating Autoimmune Diseases, CNS |  |  |  | 35 | 33 | 39 | 37 |
| Encephalomyelitis, Acute Disseminated |  |  |  | 35 | 33 | 38 | 36 |
| Felty Syndrome |  |  |  | 35 | 33 | 26 | 23 |
| Lupus Nephritis |  |  |  | 35 | 39 | 36 | 34 |
| Lupus Vasculitis, Central Nervous System |  |  |  | 35 | 33 | 35 | 33 |
| Multiple Sclerosis, Relapsing-Remitting |  |  |  | 35 | 33 | 34 | 32 |
| Myelitis, Transverse |  |  |  | 35 | 33 | 33 | 31 |
| Still's Disease, Adult-Onset |  |  |  | 35 | 33 | 30 | 28 |
| Uveomeningoencephalitic Syndrome |  |  |  | 35 | 33 | 29 | 27 |
